# Comparison of Coated and Uncoated Trace Minerals on Growth Performance, Tissue Mineral Deposition, and Intestinal Microbiota in Ducks

**DOI:** 10.3389/fmicb.2022.831945

**Published:** 2022-04-12

**Authors:** Dafei Yin, Feng Zhai, Wenbiao Lu, Amy F. Moss, Yinggu Kuang, Fangfang Li, Yujing Zhu, Ruiyang Zhang, Yong Zhang, Shuyi Zhang

**Affiliations:** ^1^College of Animal Husbandry and Veterinary Medicine, Shenyang Agricultural University, Shenyang, China; ^2^Yichun Tequ Feed Company, Yichun, China; ^3^Fujian Syno Biotech Co., Ltd., Fuzhou, China; ^4^School of Environmental and Rural Science, University of New England, Armidale, NSW, Australia

**Keywords:** duck, gut microbiota, performance, tissue deposition, trace minerals

## Abstract

Abnormally low or high levels of trace elements in poultry diets may elicit health problems associated with deficiency and toxicity, and impact poultry growth. The optimal supplement pattern of trace mineral also impacts the digestion and absorption in the body. For ducks, the limited knowledge of trace element requirements puzzled duck production. Thus, the objective of this study was to investigate the influence of dietary inclusions of coated and uncoated trace minerals on duck growth performance, tissue mineral deposition, serum antioxidant status, and intestinal microbiota profile. A total of 1,080 14-day-old Cherry Valley male ducks were randomly divided into six dietary treatment groups in a 2 (uncoated or coated trace minerals) × 3 (300, 500, or 1,000 mg/kg supplementation levels) factorial design. Each treatment was replicated 12 times (15 birds per replicate). Coated trace minerals significantly improved average daily gain (*p* < 0.05), increased Zn, Se, and Fe content of serum, liver, and muscle, increased serum antioxidant enzyme (*p* < 0.05) and decreased the excreta Fe, Zn, and Cu concentrations. Inclusions of 500 mg/kg of coated trace minerals had a similar effect on serum trace minerals and tissue metal ion deposition as the 1,000 mg/kg inorganic trace minerals. Higher concentrations of *Lactobacillus*, *Sphaerochatea*, *Butyricimonas*, and *Enterococcus* were found in birds fed with coated trace minerals. In conclusion, diets supplemented with coated trace minerals could reduce the risk of environmental contamination from excreted minerals without affecting performance. Furthermore, coated trace minerals may improve the bioavailability of metal ions and the colonization of probiotic microbiota to protect microbial barriers and maintain gut health.

## Introduction

The capacity of dietary metal ions to increase the productivity of poultry is gaining recognition. Essential trace minerals, such as zinc (Zn), iron (Fe), copper (Cu), manganese (Mn), and selenium (Se), play critical roles in the nutrition and physiology of livestock, and are provided through the diet. Abnormally low or high levels of trace elements in the body may elicit health problems associated with deficiency and toxicity, and may thereby impact poultry growth. To satisfy the trace mineral requirements of poultry, dietary supplementation of inorganic trace minerals is widely used in commercial production. This supplementation frequently oversupplies trace minerals in an attempt to avoid deficiency; however, this practice increases the excretion of minerals causing environmental contamination (Mézes et al., 2012). Yang et al. (2021) reported that avoidance of under- and overprovision of dietary minerals will achieve optimal growth performance and reduce mineral excretion. There is currently an imbalance between the development of poultry production and research in trace minerals, leading to a limited knowledge of trace mineral requirements (Bao and Choct, 2009). According to National Research Council (1994), the duck’s requirement for trace minerals was estimated based on other poultry requirements. Therefore, it is important to re-evaluate the trace mineral requirement specifically for ducks.

Furthermore, previous studies indicated that using a carbohydrate matrix to encapsulate trace minerals (coated trace minerals) protects the metal ions, reducing mineral excretion, and improving broiler growth performance (Lu et al., 2020; Yin et al., 2021). However, information on the effects of coated trace minerals in ducks is lacking.

Finally, previous reports only explore trace mineral requirements for optimal growth but do not explore the effect of trace minerals on the gut and general health of the duck (Likittrakulwong et al., 2021). Nevertheless, the concentration of minerals in tissues and blood can reflect the mineral requirements of animal (Burrow et al., 2020). Furthermore, gut microorganisms contain thousands of functionally relevant genes and pathways to support intestinal functions, protect intestinal health, and modify environmental conditions (Pajarillo et al., 2021). The competitive relationship between the host and microbiome provides an expanding field of research. Becker and Skaar (2014) reported that intestinal microbiota could inhibit host trace mineral absorption, while the host developed mechanisms to sequester trace metals and prevent bacterial access. Previous studies also showed that several gut bacteria and pathogens have the ability to transport, metabolize, and use trace minerals for survival. As Andrews et al. (2003) and Braun and Hantke (2011) reported, bacteria may bind their own or neighbor’s siderophores to transport iron across bacterial cell walls to extract metal iron concentrations. Understanding the underlying modification of trace minerals on duck intestinal microbiota is therefore important.

Therefore, the objective of this study was to investigate the effects of coated and uncoated trace minerals at three concentrations on productive performance, mineral absorption, tissue deposition, and cecal microbiota in ducks.

## Materials and Methods

### Experimental Design

The experimental design consisted of a 2 × 3 factorial arrangement with two types of trace minerals (noncoated and coated) and three levels (300, 500, and 1,000 mg/kg). The coated trace minerals were commercially produced and provided by Fujian Syno Biotech Co., Ltd.

A total of 1,080 14-day-old Cherry Valley male ducks were randomly divided into six treatment groups, each with 12 replicates of 15 ducks per replicate cage. Dietary treatments were applied from 14 to 42 days post hatch (From 1 to 13 days, birds were fed with the same commercial diets with 1,000 mg/kg trace minerals. At 14 days, the similar weight ducks were selected). The composition and nutrient levels of the experimental diets are shown in Table 1. The nutrient values met the recommendations of NY/T 2122-2012 (Ministry of Agriculture of the People’s Republic of China, 2012). Trace mineral content of diets are given in Supplementary Table S1. The ducks were reared in cages (2.2 m × 1.2 m × 0.9 m) in a temperature- and humidity-controlled room and allowed *ad libitum* access to feed and water. Total feed consumption and body weight for each replicate cage of birds were recorded at 14 and 42 days post-hatch. Average daily feed intake (ADFI), average daily weight gain (ADG) in weight, and feed conversion ratio (FCRs) were determined over the experimental period.

**Table 1:** Feed ingredients and nutrient composition of experimental diets.

Ingredients	%	Nutrient levels^2^	%
Corn	51.45	ME/(MJ/kg)	12.93
Soybean meal	19.20	Crude protein	19.62
Soy oil	3.00	Calcium	0.79
Corn DDGS	11.40	Available phosphorous	0.40
Corn gluten meal	4.50		
Dicalcium phosphate	1.20		
Polishing powder	5.00		
NaCl	0.25		
Feather meal	1.00		
Glutamic acid residue	2.00		
Premix^1^	1.00		

1 *The premix provided the following nutrients per kg of diet: VA1, 500 IU, VE 25 mg, VD 4,000 IU, VK 2 mg, VB1 1.5 mg, VB2 6 mg, VB6 4 mg, VB12 0.02 mg, biotin 0.15 mg, pantothenic acid 11 mg, folic acid 0.5 mg, nicotinamide 40 mg, lysine 450 mg, methionine 250 mg, threonine 100 mg, and phytase 10 mg*.

2 *Nutritional levels are calculated values*.

### Sample Collection

On day 42, six birds per treatment were randomly selected, and blood samples were drawn from the brachial vein. Serum samples were centrifuged at 1,500 × *g* at 4°C for 20 min and stored at −20°C until further analysis. Then the selected birds were euthanized by intravenous administration of sodium pentobarbital (50 mg/kg of body weight) into the wing vein. One lobe of the liver and breast muscle samples were immediately frozen in liquid nitrogen and stored at −80°C before analysis.

### Nutrient Digestibility

For the determination of apparent crude protein, crude fat, calcium, and phosphorus digestibility, excreta from each pen was collected and weighed for a 78-h period from day 39 to day 42 post-hatch and dried for analysis and to determine excreta dry matter. Feed and excreta samples were then ground to pass through a 40-mesh screen and analyzed for crude protein (CP; method 990.03; AOAC, 2000), ether extract (EE; method 920.39; AOAC, 2000) and crude fiber (*CF*; method 978.10; AOAC, 2000). Calcium and phosphorus determination was carried out according to the method of David et al. (2019).

### Mineral Concentration

A quarter of the total feces content collected was dried at 65°C for 48 h, broken up, and sifted to remove feathers. Then the fecal sample was ground to pass through a 1-mm screen after samples achieved constant weight and packed in self-locked plastic bags and stored at −20°C for analysis. Liver and breast muscle samples were freeze dried and finely ground using a 1-mm screen. Serum sample was deproteinated by 10% (w/v) trichloracetic acid. The Fe, Mn, Zn, and Cu concentrations in fecal samples, tissues, and serum were analyzed using an atomic absorption spectrophotometer (Z-8200, Hitachi, Tokyo, Japan). Selenium concentration was analyzed after wet ashing with nitric and perchloric acids and measured by the fluorometric method (method 996.16; AOAC, 2000).

### Antioxidant Indices

The activity of Cu/Zn superoxide dismutase (Cu/Zn-SOD), glutathione peroxidase (GSH-Px), malondialdehyde (MDA), and total antioxidant capacity (T-AOC) content in serum were determined using the colorimtric method, TBA method, an r-911 automatic radioactivity meter (7,160, Hitachi, Japan), and light amide method, respectively, following the instructions of the manufacturer (Jiangsu Baolai Biotechnology Co. Ltd.).

### Analysis of the Cecal Microbiota Community by 16S rRNA Analysis

The cecal contents from 12 birds were collected, then mixed in pairs, and treated as one sample. Cecal digesta were immediately frozen in liquid nitrogen and stored at −80°C for DNA isolation. The total genome DNA from the cecal samples was extracted using QIAamp DNA stool Mini Kit (Qiagen, Germany) according to the protocol of the manufacturer. DNA was quantified in a NanoDrop ND-1000 spectrophotometer (Thermo Scientific, Waltham, MA, United States) and stored at −80°C until processed for amplification. The construct 16S rRNA sequencing libraries, the V3–V4 regions of the 16S rRNA gene, were amplified from the DNA samples by PCR using the primer set of 338F (5′-ACTCCTACGGGAGGCAGCAG-3′) and 806R (5′-GGACTACHVGGGTWTCTAAT-3′) by the thermocycler PCR system (GeneAmp 9,700, ABI, United States). PCR reactions were performed in triplicate in a 20-μl mixture containing 4 μl of 5× FastPfu Buffer, 2 μl of 2.5 mM dNTPs, 0.8 μl of each primer (5 μM), 0.4 μl of FastPfu Polymerase, and 10 ng of template DNA. The PCR reactions were conducted using the following program, 3 min of denaturation at 95°C, 30 cycles of 30 s at 95°C, 30 s of annealing at 55°C, and 45 s of elongation at 72°C, and a final extension at 72°C for 10 min. The resulting PCR products were extracted from a 2% agarose gel and further purified using the AxyPrep DNA Gel Extraction Kit (Axygen Biosciences, Union City, CA, United States) and quantified using QuantiFluor™^-ST^ (Promega, United States) according to the protocol of the manufacture. Finally, the libraries were sequenced on an Illumina Hiseq 2500 platform, and 250-bp paired-end reads were generated.

### Sequence Processing

Raw data FASTQ files were imported into the format, which could be operated by QIIME2 system using QIIME tools import program. Demultiplexed sequences from each sample were quality filtered and trimmed, de-noised, merged, and then the chimeric sequences were identified and removed using the QIIME2 dada2 plugin to obtain the feature table of amplicon sequence variant (ASV; Callahan et al., 2016). The QIIME2 feature-classifier plugin was then used to align ASV sequences to a pre-trained GREENGENES 13_8 99% database to generate the taxonomy (Bokulich et al., 2018). As usual, the ASV with 99% similarity was classified as one operational taxonomic unit (OTU) to obtain the OTU classification information.

### Bioinformatics Analysis

The alpha-diversity analysis was calculated, including microbial community richness parameters (Chao1), and community diversity parameters (Shannon diversity index and Simpson indices). The differences among treatments for α-diversity was calculated according to a Kruskal–Wallis multiple comparison followed by Wilcoxon’s pairwise comparison for significantly different groups. Beta-diversity distance measurements were calculated and visualized *via* principal coordinate analysis (PCoA). Similarity analysis (permutation multivariate analysis, PERMANOVA; Analysis of similarities, ANOSIM) tests were conducted based on beta diversity.

To identify differential microbial species among the different treatments, the linear discriminant analysis and effect size (LEfSe) analysis based on the linear discriminant analysis (LDA) were used, and the significant differences with an LDA score >2.0 as the critical value were assessed. The redundancy analysis method (RDA) and Spearman’s analysis were used to analyze the potential association between the intestinal flora and environmental variables based on relative abundances of microbial species at order and genus levels using the R package “vegan.”

### Function Analysis

The potential Kyoto Encyclopedia of Genes and Genomes (KEGG) functional profiles of microbial communities were predicted with PICRUSt2 and annotated by the MetaCyc and ENZYME database. In KEGG level 3 pathway analysis, the enrichment of each pathway was converted to a Z-score.

### Statistical Analysis

Data derived from ducks in 12 replicate cages per treatment were analyzed as a 2 × 3 factorial arrangement (two-way analysis of variance) using IBM SPSS Statistics 20.0 (IBM Corporation, Somers, NY, United States). Probability values *p* ≤ 0.05 were considered significant.

## Results

### Growth Performance

The influence of dietary treatments on growth performance is shown in Table 2. Trace minerals significantly affected ADG (*p* < 0.05), wherein the coated trace minerals had a more pronounced effect than uncoated minerals, and ADG increased with greater supplementation of trace minerals. Dietary treatments had no significant influence on ADFI and FCR. There was no significant interaction between dietary supplementation pattern and supplementation level of trace minerals on growth performance.

**Table 2:** Effects of trace mineral type (coated or uncoated) and supplementation level (300, 500, or 1,000 mg/kg) of trace minerals on the growth performance of ducks.

Trace minerals	Level (mg/kg)	ADFI, g/d	ADG, g/d	FCR
Uncoated	300	207.38	95.40	2.17
	500	219.13	97.36	2.25
	1,000	215.93	101.74	2.12
Coated	300	212.45	96.89	2.19
	500	207.30	99.71	2.08
	1,000	218.69	103.74	2.11
SEM		7.500	1.870	0.062
*Main effect*				
Trace minerals	Coated	212.81	100.12	2.13
	Uncoated	214.14	98.17	2.18
Level	300	209.92	96.15^c^	2.18
	500	213.21	98.54^b^	2.16
	1,000	217.31	102.74^a^	2.12
*Value of* *p*				
Trace minerals		0.712	0.031	0.147
Level		0.261	<0.001	0.334
Interaction		0.138	0.914	0.108

*ADFI, average daily feed intake; ADG, average daily weight gain; FCR, feed conversion ratio*.

a,b,c *Means within columns not sharing a common superscript are significantly different at the 5% level of probability*.

### Nutrient Digestibility

The influence of dietary treatments on total tract nutrient digestibility in ducks at 42 days is shown in Table 3. There was a significant main effect where coated trace minerals significantly increased phosphorus digestibility compared with uncoated trace minerals. Additionally, CP digestibility in birds fed with 1,000 mg/kg of trace minerals was higher than those fed with 300 and 500 mg/kg. While not significantly different, there is a tendency that 300 mg/kg of trace minerals had a lower EE (*p* = 0.051) and CF (*p* = 0.088) digestibility than 500 and 1,000 mg/kg of trace minerals.

**Table 3:** Effects of trace mineral type (coated or uncoated) and supplementation level (300, 500, or 1,000 mg/kg) of trace minerals on the total tract nutrient digestibility of ducks.

Trace minerals	Level (mg/kg)	DM	CP	EE	Ca	P	CF
Uncoated	300	69.00	50.81	75.71	36.05	43.26	35.12
	500	70.73	50.26	77.57	36.14	42.24	36.63
	1,000	70.00	51.90	77.22	35.42	41.43	36.96
Coated	300	69.35	50.30	75.72	37.60	45.86	35.58
	500	69.45	51.56	77.69	35.25	43.85	35.89
	1,000	71.14	52.23	77.15	37.64	43.00	37.55
SEM		0.968	0.613	1.160	1.205	1.725	1.268
*Main effect*							
Trace minerals	Coated	69.98	51.36	76.85	36.83	44.24	36.34
	Uncoated	69.91	50.99	76.83	35.87	42.31	36.24
Level	300	69.18	50.56^b^	75.71	36.83	44.56	35.35
	500	70.09	50.91^b^	77.63	35.70	43.05	36.26
	1,000	70.57	52.06^a^	77.19	36.53	42.21	37.26
*Value of* *p*							
Trace minerals		0.891	0.289	0.973	0.136	0.044	0.872
Level		0.114	0.008	0.051	0.319	0.118	0.088
Interaction		0.179	0.137	0.991	0.128	0.859	0.651

a,b *Means within columns not sharing a common superscript are significantly different at the 5% level of probability*.

### Tissue Mineral Deposition

Table 4 shows the influence of dietary treatments on liver mineral deposition. There was a significant interaction of liver Se and Mn content. The supplementation of 500 mg/kg of coated trace minerals had the highest liver Se concentration among the dietary treatments. The highest liver Mn concentration was found in 1,000 mg/kg of coated trace minerals. Liver Zn and Cu significantly increased with greater supplementation of trace minerals. Birds fed with 1,000 mg/kg of trace minerals had the lowest liver Fe content. There is a tendency that birds fed with coated trace minerals had an increased liver Zn (*p* = 0.053) and Fe (*p* = 0.071) content.

**Table 4:** Effects of trace mineral type (coated or uncoated) and supplementation level (300, 500, or 1,000 mg/kg) of trace minerals on liver mineral deposition in ducks (mg/kg).

Trace minerals	Level (mg/kg)	Se	Zn	Fe	Cu	Mn
Uncoated	300	0.60^c^	47.97	298.67	58.00	3.50^b^
	500	0.59^c^	71.17	336.50	67.38	4.05^b^
	1,000	0.87^a^^,^^b^	81.9	317.33	80.00	4.50^b^
Coated	300	0.78^b^	58.62	345.67	65.75	3.92^b^
	500	1.02^a^	80.88	367.33	72.93	4.10^b^
	1,000	0.83^b^	86.45	304.83	90.13	5.63^a^
SEM		0.143	11.605	33.965	18.208	0.833
*Main effect*						
Trace minerals	Coated	0.88	75.32	339.28	76.27	4.55
	Uncoated	0.69	67.01	317.5	68.46	4.01
Level	300	0.69^b^	53.29^b^	322.17^a^	61.88^b^	3.71^b^
	500	0.81^a^^,^^b^	76.03^a^^,^^b^	351.92^a^	70.16^a^^,^^b^	4.08^b^
	1,000	0.85^a^	84.18^a^	311.08^b^	85.07^a^	5.06^a^
*Value of* *p*						
Trace minerals		<0.001	0.053	0.071	0.226	0.079
Level		0.041	<0.001	0.021	0.018	0.002
Interaction		0.002	0.810	0.114	0.957	0.002

*Se, selenium; Zn, zinc; Fe, iron; Cu, copper; and Mn, manganese*.

a,b,c *Means within columns not sharing a common superscript are significantly different at the 5% level of probability*.

Table 5 shows the influence of dietary treatments on mineral deposition in muscle.

**Table 5:** Effects of trace minerals type (coated or uncoated) and supplementation level (300, 500, or 1,000mg/kg) of muscle mineral deposition in ducks.

Trace minerals	Level (mg/kg)	Se (μg/kg)	Zn (mg/kg)	Fe (mg/kg)	Cu (mg/kg)	Mn (μg/kg)
Uncoated	300	76.67^c^	14.13	43.35	1.92	249.00
	500	82.17^c^	14.97	41.90	2.48	274.17
	1,000	131.67^a^	17.70	47.85	2.80	275.17
Coated	300	109.00^b^	16.83	47.10	2.15	250.00
	500	138.33^a^	17.00	51.63	3.29	284.83
	1,000	124.83^a^	19.28	47.00	2.86	304.83
SEM		12.037	3.097	5.323	1.217	57.705
*Main effect*						
Trace minerals	Coated	124.06	17.71	48.58	2.77	279.89
	Uncoated	96.83	15.60	44.37	2.40	266.11
Level	300	92.83^c^	15.48^b^	45.23	2.04	249.50
	500	110.25^b^	15.98^b^	46.77	2.88	279.50
	1,000	128.25^a^	18.49^a^	47.43	2.83	290.00
*Value of* *p*						
Trace minerals		<0.001	0.004	0.026	0.421	0.506
Level		<0.001	0.001	0.597	0.240	0.260
Interaction		<0.001	0.842	0.071	0.777	0.845

a,b,c *Means within columns not sharing a common superscript are significantly different at the 5% level of probability*.

There is a significant interaction for muscle Se content where 500 mg/kg of coated trace minerals generated the highest muscle concentration of Se. Coated trace minerals generated the highest muscle concentrations of Zn and Fe, and 1,000 mg/kg of trace mineral inclusion generated the highest Zn concentration in muscle.

### Fecal Mineral Contents

The effects of dietary treatments on fecal mineral excretion are presented in Table 6. There was a significant interaction for Fe, where 1,000 mg/kg of uncoated trace mineral generated the greatest amount of Fe in excreta, whereas 300 mg/kg of uncoated trace mineral generated the lowest. Se, Zn, and Mn concentrations in feces increased with greater supplementation of trace minerals. Uncoated trace minerals generated greater concentration of Zn in excreta, but coated trace minerals generated a greater concentration of Mn within excreta.

**Table 6:** Effects of trace mineral type (coated or uncoated) and supplementation level (300, 500, or 1,000 mg/kg) of trace minerals on fecal excretion ion metals contents in ducks (mg/kg).

Trace minerals	Level (mg/kg)	Se	Zn	Fe	Cu	Mn
Uncoated	300	0.56	3.00	7.80^c^^,^^d^	0.33	2.43
	500	0.91	3.37	9.47^b^	0.33	3.40
	1,000	1.16	3.63	14.2^a^	0.40	3.27
Coated	300	0.49	2.53	7.13^d^	0.27	2.00
	500	0.81	2.87	8.20^c^	0.37	2.50
	1,000	1.08	3.30	8.23^c^	0.4	2.90
SEM		0.118	0.378	0.503	0.057	0.147
*Main effect*						
Trace minerals	Coated	0.79	2.9	7.86	0.34	2.47
	Uncoated	0.88	3.33	10.49	0.36	3.03
Level	300	0.52^c^	2.77^b^	7.47^c^	0.30	2.22^b^
	500	0.86^b^	3.12^a^^,^^b^	8.83^b^	0.35	2.95^a^
	1,000	1.12^a^	3.47^a^	11.22^a^	0.40	3.08^a^
*Value of* *p*						
Trace minerals		0.224	0.038	<0.001	0.712	<0.001
Level		<0.001	0.031	<0.001	0.051	<0.001
Interaction		0.976	0.928	<0.001	0.397	0.057

a,b,c,d *Means within columns not sharing a common superscript are significantly different at the 5% level of probability*.

### Serum Mineral Levels

The effects of dietary treatments on serum Se, Zn, Fe, Cu, and Mn concentrations are given in Table 7. There was a significant interaction where 500 mg/kg of coated trace minerals generated the greatest concentration of Se in serum. Zn and Fe serum concentrations were increased with greater trace mineral supplementation. Coated trace minerals increased Zn in serum.

**Table 7:** Effects of trace mineral type (coated or uncoated) and supplementation level (300, 500, or 1,000 mg/kg) of trace minerals on serum ion metals contents in ducks.

Trace minerals	Level (mg/kg)	Se (μg/ml)	Zn (μg/ml)	Fe (μg/ml)	Cu (ng/ml)	Mn (μg/ml)
Uncoated	300	142.17^c^	1.97	129.03	135.02	6.79
	500	164.81^b^	2.10	156.49	147.07	7.41
	1,000	185.52^a^	2.15	151.15	148.94	8.05
Coated	300	151.54^b^^,^^c^	2.14	137.43	138.40	6.89
	500	197.07^a^	2.26	154.56	138.18	8.03
	1,000	193.79^a^	2.35	148.21	157.20	8.13
SEM		10.767	0.152	20.137	21.028	1.763
*Main effect*						
Trace minerals	Coated	180.80	2.25	146.73	144.59	7.68
	Uncoated	164.17	2.07	145.56	143.68	7.42
Level	300	146.86^b^	2.06^b^	133.23^b^	136.71±	6.84
	500	180.94^a^	2.18^a^^,^^b^	155.53^a^	142.63	7.72
	1,000	189.65^a^	2.25^a^	149.68^a^^,^^b^	153.07	8.09
*Value of* *p*						
Trace minerals		<0.001	0.002	0.762	0.899	0.688
Level		<0.001	0.022	0.027	0.187	0.285
Interaction		<0.001	0.973	0.433	0.608	0.929

a,b,c *Means within columns not sharing a common superscript are significantly different at the 5% level of probability*.

### Antioxidant Status

The effects of dietary treatments on serum antioxidant status are shown in Table 8. Significant interactions were found in serum MDA content and GSH-Px activity where ducks offered 500 mg/kg of coated trace minerals resulted in the lowest serum MDA content and highest serum GSH-Px activity. Coated trace minerals and the increasing supplementation of dietary trace minerals significantly improved serum SOD activities. Dietary treatments had no significant influence on serum T-AOC activity.

**Table 8:** Effects of trace mineral type (coated or uncoated) and supplementation level (300, 500, or 1,000 mg/kg) of trace minerals on serum antioxidant status in ducks.

Trace minerals	Level (mg/kg)	MDA, (nmol/ml)	SOD (pg./ml)	GSH-Px, (pg./ml)	T-AOC, (U-pg/ml)
Uncoated	300	15.21^a^	305.30	293.30^d^	12.68
	500	13.79^b^	418.88	328.73^d^	13.51
	1,000	9.09^d^	505.25	416.72^b^	14.77
Coated	300	11.73^c^	381.03	375.03^c^	13.35
	500	9.05^d^	442.75	475.82^a^	14.38
	1,000	9.93^d^	555.05	392.23^b^^,^^c^	15.29
SEM		0.900	41.018	30.112	2.263
*Main effect*					
Trace minerals	Coated	10.24	459.61	410.75	14.28
	Uncoated	13.01	404.05	348.44	13.51
Level	300	14.44^a^	346.61^c^	334.17^b^	13.01
	500	10.39^b^	430.82^b^	410.44^a^	13.94
	1,000	9.59^c^	535.13	404.48^a^	15.06
*Value of* *p*					
Trace minerals		<0.001	0.002	<0.001	0.406
Level		<0.001	<0.001	<0.001	0.16
Interaction		<0.001	0.348	<0.001	0.984

*MDA, malondialdehyde; SOD, superoxide dismutase; GSH-Px, glutathione peroxidase; and T-AOC, total antioxidant capacity*.

a,b,c,d *Means within columns not sharing a common superscript are significantly different at the 5% level of probability*.

### Alpha-Diversity

Dietary treatments had significant interactions for Chao1, Shannon, and Simpson indices (Table 9). Ducks fed with 500 mg/kg of uncoated trace mineral had the highest Chao1, Shannon, and Simpson indices, and the 1,000 mg/kg of uncoated trace mineral treatment resulted in the lowest Chao 1 and Shannon indices. Diets supplemented with 300 mg/kg of uncoated trace minerals significantly decreased Simpson index compared with the other dietary treatments.

**Table 9:** Effects of trace mineral type (coated or uncoated) and supplementation level (300, 500, or 1,000 mg/kg) of trace minerals on duck cecal microbiota ⍺-diversity.

Trace minerals	Level (mg/kg)	Chao1	Shannon	Simpson
Uncoated	300	423.83^a^	6.93^a^^,^^b^	0.97^b^
	500	453.83^a^	7.46^a^	0.99^a^
	1,000	323.67^b^	6.53^b^	0.98^a^^,^^b^
Coated	300	385.00^a^^,^^b^	7.16^a^^,^^b^	0.98^a^^,^^b^
	500	364.33^a^^,^^b^	6.96^a^^,^^b^	0.98^a^^,^^b^
	1,000	412.00^a^^,^^b^	7.12^a^^,^^b^	0.98^a^^,^^b^
SEM		65.578	0.437	0.007
*Main effect*				
Trace minerals	Coated	387.11	7.08	0.98
	Uncoated	400.44	6.97	0.98
Level	300	404.42	7.04	0.98
	500	409.08	7.21	0.98
	1,000	367.83	6.83	0.98
*Value of* *p*				
Trace minerals		0.569	0.524	0.614
Level		0.296	0.183	0.316
Interaction		0.011	0.036	0.031

a,b *Means within columns not sharing a common superscript are significantly different at the 5% level of probability*.

### Microbial Composition

**Figure 1:**
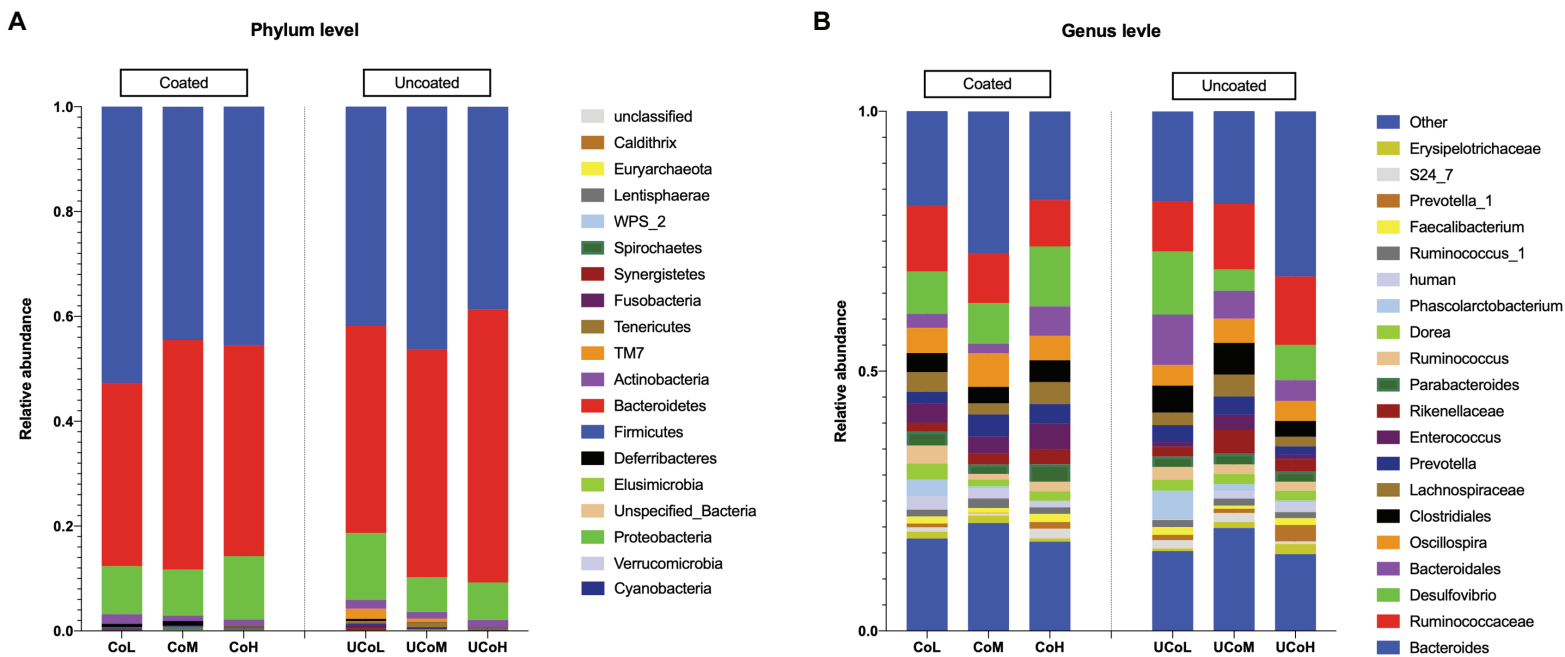
Differentially abundant taxa between diets supplemented with different levels of coated and uncoated trace minerals. **(A)** Represented phylum level, **(B)** represented top 20 genera. Data are means of six replicates. CoL, 300 mg/kg of coated trace mineral treatment; CoM, 500 mg/kg of coated trace mineral treatment; CoH, 1,000 mg/kg of coated trace mineral treatment; UCoL, 300 mg/kg of uncoated trace minerals treatment; UCoM, 500 mg/kg of uncoated trace minerals treatment; and UCoH, 1,000 mg/kg of uncoated trace minerals treatment.

The taxonomic distribution of the gut microbiota in treatments is shown in Figure 1. At phyla level, Firmicutes, Bacteroidetes, and Proteobacteria were dominant (>1%). Coated trace mineral increased Firmicutes abundance in cecum of ducks. The top five relative abundances at phylum level in each treatment were Firmicutes, Bacteroidetes, Proteobacteria, Actinobacteria, and Cyanobacteria (Figure 1A). Ducks that received coated trace minerals showed a reduction in Tenericutes. As shown in Supplementary Figure S1, the dominant families in the sample were *Bacteroidaceae* and *Ruminococcaceae*, followed by *Desulfovibrionaceae*, *Lachnospiraceae*, and *Bacteroidales* in each treatment. At the genus level, the top 20 genera are listed in Figure 1B. *Bacteroides* was the most abundant genus at 18% of the sequence of all dietary treatments. There was an increase in the abundance of *Oscillospira* and *Enterococcus* when birds were fed with coated trace minerals. The *Ruminococcus* and *Phascolarctobacterium* were identified in high abundance in lowest trace minerals supplement groups, while *Prevotella_1* was identified in high abundance (>0.01%) when ducks were offered the highest level of trace minerals.

### Beta Diversity

**Figure 2:**
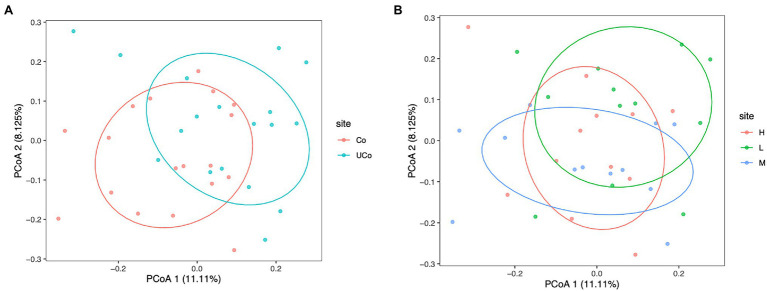
Principal coordinate analysis (PCoA) of cecal samples based on the unweighted UniFrac Distances. **(A)** The labels of the samples indicate the pattern of trace minerals, Co, coated trace minerals; UCo, uncoated trace minerals. **(B)** The labels of the samples indicate the supplementation level of trace minerals, L, 300 mg/kg; M, 500 mg/kg; and H, 1,000 mg/kg.

In the present study, beta diversity was calculated to evaluate the similarity of the microbial community structures. Principal component analysis (PCoA) based on the unweighted UniFrac Distances was employed (Figure 2). The PCoA results showed that the cecal microbiota was barely separated from that in coated and uncoated trace mineral treatments (PERMANOVA, *p* = 0.001; ANOSIM, *R* = 0.200, *p* = 0.001; Figure 2A), while there is also no significant separation between different levels of trace minerals on duck microbiota flora (ANOSIM analysis: L/M: *R* = 0.207, *p* = 0.002; L/H: *R* = 0.207, =0.005; M/H: *R* = 0.096, *p* = 0.04; Figure 2B).

**Figure 3:**
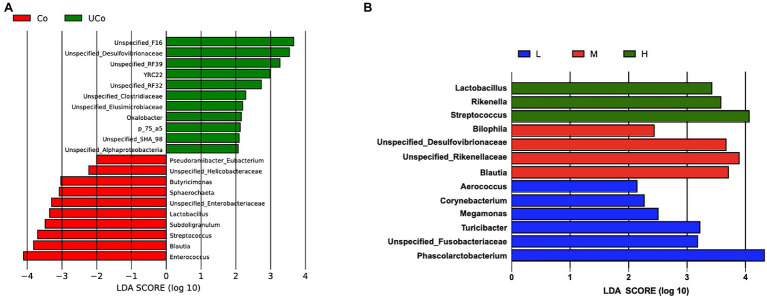
Effect size (LEfSe) analysis based on genus level between treatments. **(A)** Co, coated trace minerals (red); UCo, uncoated trace minerals (green); **(B)** L 300 mg/kg (blue), M, 500 mg/kg (red); and H, 1,000 mg/kg (green).

By LEfSe analysis, there were 21 differentially abundant bacterial clades at genus level (LDA score >2.0) between the coated and uncoated trace mineral treatments (Figure 3). *Lactobacillus*, *Sphaerochatea*, *Butyricimonas*, and *Enterococcus* were the dominant genus in cecum when ducks were fed with coated trace minerals, while the uncoated trace mineral-treated bird cecum were inhabited mostly by *Desulfovibronaceae*, *Clostridiaceae*, *Elusimicrobiaceae*, and *Oxalobacter*. Alternatively, 13 differentially abundant microbiota were found in three supplementation levels of trace mineral treatments. The 1,000 mg/kg of trace minerals could improve *Lactobacillus*, *Rikenella*, and *Streptococcus* colonization in duck cecum, and *Biophila*, *Blautia*, *Rikenellaceae*, and *Desulfovibrionaceae* were the dominant genus in 500-mg/kg treatments. When diets were supplemented with 300 mg/kg of trace minerals, *Phascolarctobacterium*, *Turicibacter*, *Megamonas*, and *Corynebacterium* were enriched in duck cecum.

### Correlations Between Fecal Metal Concentrations and Gut Microbiota

**Figure 4:**
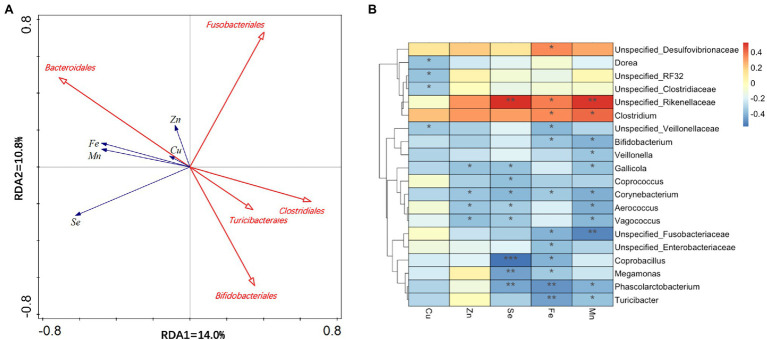
Correlation between fecal metal concentration and gut microbiota at order level. **(A)** Redundancy analysis (RDA) plot showed significant (*p* < 0.05) relationships between environmental variables (fecal metal concentration) and cecal bacterial communities. **(B)** Spearman’s heatmap correlation on order combined with UPGMA cluster analysis between abundance of series of to 25 bacteria order and environmental parameters. Negative correlation and positive correlation were represented by blue color and red color. ^*^0.01 < *p* ≤  0.05, ^**^0.001 < *p* ≤  0.01, and ^***^p ≤  0.001.

A multivariate analysis was carried out using the fecal metal concentrations Se, Zn, Fe, and Cu as “environmental variables” and the order groups as “species variable.” RDA showed that a total of 25% of the total variation in the microbiota composition at order level is related to environment variables (fecal metal concentrations). A smaller angle represents a positive relationship. In contrast, an angle more than 90° indicates a negative relationship. The arrow length reflects the importance of the parameter, and the orientation represents the association between the parameter and the axis. Five significant correlated orders are shown in Figure 4A, and among these microbiotas, there is a positive relationship between cecal *Bacteroidals* and fecal metal concentrations. *Fusobacterialse* has a negative relationship with fecal Zn concentration. Duck cecal *Clostridiales*, *Turicibacterales*, and *Bifidobacteriales* had negative relationships with fecal trace mineral contents. Additionally, fecal Se concentration had an important influence on excreta metal ion concentrations.

Spearman’s correlation analysis of fecal Se, Zn, Fe, Cu, and Mn, and the most abundant 20 bacterial genera among the cecal microbiota was performed. As shown in Figure 4B, positive correlations (correlation coefficient >0) and negative correlations (correlation coefficient <0) were observed between various bacteria and environmental factors. An extreme negative correlation was found between cecal *Coprobacillus* and fecal Se concentration.

### Functional Predictions Based on 16S rRNA Gene Sequence

**Figure 5:**
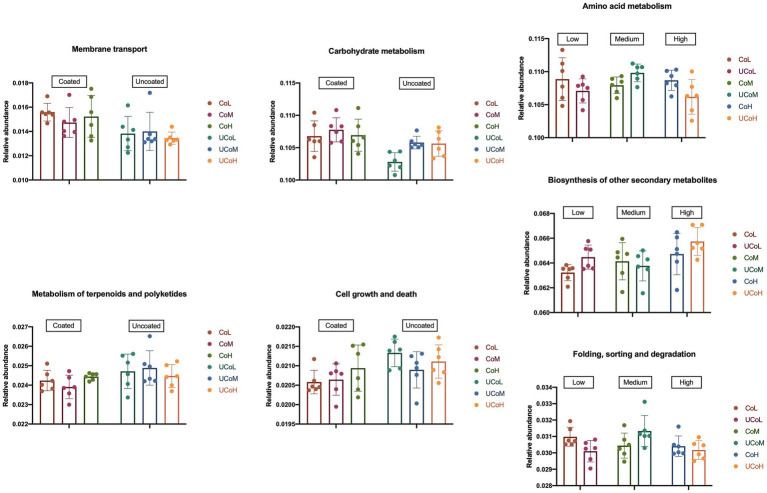
Comparison of microbiota function related to Kyoto Encyclopedia of Genes and Genomes (KEGG) pathway at level 2. CoL, 300 mg/kg of coated trace mineral treatment; CoM, 500 mg/kg of coated trace mineral treatment; CoH, 1,000 mg/kg of coated trace mineral treatment; UCoL, 300 mg/kg of uncoated trace minerals treatment; UCoM, 500 mg/kg of uncoated trace minerals treatment; and UCoH, 1,000 mg/kg of uncoated trace minerals treatment.

**Figure 6:**
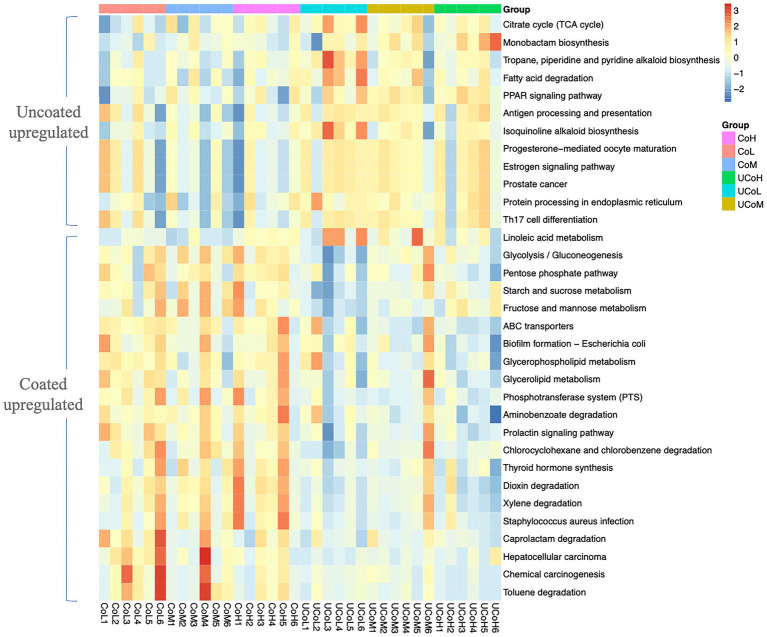
Comparison of microbiota function related to KEGG pathway at level 3. CoL, 300 mg/kg of coated trace mineral treatment; CoM, 500 mg/kg of coated trace mineral treatment; CoH, 1,000 mg/kg of coated trace mineral treatment; UCoL, 300 mg/kg of uncoated trace minerals treatment; UCoM, 500 mg/kg of uncoated trace minerals treatment; and UCoH, 1,000 mg/kg of uncoated trace minerals treatment.

According to KEGG level 2 analysis (Figure 5), coated trace mineral treatments were significantly enriched in the membrane transport and carbohydrate metabolism, while uncoated trace mineral groups were enriched in metabolism of terpenoids, polyketides, cell growth, and death pathways. Furthermore, supplementation levels of trace minerals had a significant influence on amino acid metabolism, biosynthesis of other secondary metabolites, and folding, sorting, and degradation pathways. At KEGG level 3 (Figure 6), we found 33 pathways that were significantly different between coated and uncoated trace mineral treatments. Among them, coated trace mineral groups significantly upregulated 20 pathways including glycolysis, pentose phosphate pathway, and glycerolipid metabolism. Uncoated trace mineral groups were enriched in 13 pathways, such as TCA cycle, fatty acid degradation, and linoleic acid metabolism.

## Discussion

### Growth Performance and Nutrient Digestibility

The present study aimed to determine the effect of coated and uncoated trace minerals on duck performance, mineral absorption, tissue deposition, and cecal microbiota. It has been clearly established that dietary trace metals can influence duck ADG during the 14- to 42-day feeding period. Diets containing coated trace minerals had superior ADG in weight compared with those containing uncoated trace minerals. Despite the fact that there is little information about the effect of coated trace minerals on duck performance, it is possible that controlling the release of trace metals in the gastrointestinal tract could improve the efficacy of ion metal absorption (Lei and Kim, 2018), and thus duck growth. Villagómez-Estrada et al. (2020) and Shannon and Hill (2019) reported that the primary functions of trace elements are to be part of a large number of coenzymes for various biological processes. Additionally, previous studies have proven that trace minerals play an essential role in digestive enzyme activities in pancreatic tissue and small intestine, such as Zn, Fe, and Mn (Alkhtib et al., 2020). The present study agrees with previous reports in pigs and broilers, where coating technology acted as a performance enhancer owing to higher bioavailability (Lei and Kim, 2018; Khatun et al., 2019; Yin et al., 2021). The cause behind the observed increase in ADG associated with feeding coated trace minerals appears to be an improved mineral utilization. Alternatively, it has been proposed that the positive effect of coated Zn supplementation on broiler performance might be associated with the improvement of appetite, altered growth hormone production, and enhanced capacity for immune response (Alkhtib et al., 2020). The observed higher phosphorus digestibility found in birds fed with coated trace minerals might be associated with high gastric pH values inhibiting the antagonisms between microminerals and phytic acid to improve the absorption and bioavailability (Liu et al., 2014).

### Deposition of Minerals

Blood mineral content may reflect the mineral bioavailability (Burrow et al., 2020). The present study found that serum Se and Zn concentrations were improved with coated micromineral supplementation. Dietary inclusion of 1,000 mg/kg of trace minerals had an incremental effect on serum Se and Zn, which is in agreement with previous results (Zhang et al., 2017; Olukosi et al., 2018). Metal ions coated with carbohydrate might alter the ion interaction affecting the antagonism, which might potentially improve the utilization of the minerals or affect the ligand resulting in alteration in their utilization (Wang et al., 2019b). The interaction between trace mineral type and supplementation level in serum Se content suggested that dietary inclusion with 500 mg/kg of coated trace minerals had potential ability to replace 1,000 mg/kg of inorganic trace mineral in duck diets.

Excess intake of dietary metal ions leads to the higher deposition in the liver (Mézes et al., 2012). Thus, it is sensible that in the present study, with the exception of Fe, diets supplemented with the highest dosage of trace minerals resulted in the highest liver ion concentrations. Liver Se was also increased under coated trace mineral treatments. Coated trace minerals might potentially result in unique and more stable structures, which facilitate access to the enterocyte and improve the deposition in tissues (Bai et al., 2017; Wang et al., 2019b). Additionally, Se, Fe, and Zn deposition in muscle were significantly increased in birds fed with coated trace minerals. Qiu et al. (2020) reported that metal ions, such as Fe could be easily stored in tissues, and their absorption rate could be altered by adjusting the amount and form in the feed. The present study suggested that diets supplemented with 500 mg/kg of coated trace minerals were more efficient than the same or higher level of inorganic trace minerals, since coated forms are more conducive to the deposition of Se in the body.

Lower efficiency of utilization occurring with high supplementation of minerals resulted in greater excretion, which can be assumed to produce greater environmental pollution. Creech et al. (2004) indicated that reducing dietary trace mineral level could reduce excretion of minerals in swine waste. The present result found that coated trace minerals significantly reduced fecal Zn, Fe, and Mn contents, which agrees with previous studies. This suggests that the environmental impact of trace mineral supplementation may be mitigated by using coated trace minerals, which are demonstrated to be more bioavailable in the present study.

### Antioxidant Status

Damage from reactive oxygen species could be reduced by SOD and GSH-Px (Wang et al., 2019a). These two enzymes are related with Zn, Cu, and Se concentration. MDA is also a marker of lipid peroxidation (Samuel et al., 2017). The present study found that coated trace minerals improved serum antioxidant status, which was similar with a previous report (Yin et al., 2021). The coated form of trace minerals may provide benefits through modulating the antioxidant system owing to resistant antagonisms in the intestine. We also found that ducks offered with 500 mg/kg of coated trace mineral had a similar antioxidant status than those offered with 1,000 mg/kg of inorganic trace minerals, suggesting that the effect of coated trace minerals is greater or equal compared with that of inorganic trace minerals.

### Microbiota Profile

The gut microbiota contains thousands of functionally relevant genes and pathways to support essential gut functions and prevent intestinal dysbiosis (Pajarillo et al., 2021). Under a deficiency of trace minerals, gut microbiota would rapidly mature to respond to this stress (Bao et al., 2010; Tako et al., 2011). Reed et al. (2015) reported that the insufficient bioavailable dietary Zn in the lumen might improve gut microbiota colonization. Thus, the higher microbiota α-diversity associated with rapid proliferation in birds fed with inorganic trace minerals might reflect that absorption of inorganic trace mineral supplementation earlier in the intestine may result in a lack of trace minerals in the cecum. The overall microbial structure between coated and uncoated trace mineral treatments showed that trace minerals significantly influenced the microbiota profile in duck cecum. From LEfSe analysis, *Lactobacillus*, *Sphaerochaetea*, *Butyricimonas*, and *Enterococcus* were predominant genus enriched in birds fed with coated trace minerals. However, *Desulfovibronaceae* was enriched in inorganic trace mineral treatments. *Desulfovibronaceae* is a member of sulfate-reducing bacteria family, which potentially produced endotoxin, leading to chronic inflammation (Zhang-Sun et al., 2015). Alternatively, coated trace minerals could improve the probiotic microbiota colonization. *Sphaerochaeta* is both fermenter and electrogenic bacteria, which might play an important role in substrate transformation and electricity generation (Li et al., 2020), and higher enrichment of *Sphaerochaeta* is associated with lower ion excreta in coated trace mineral treatments. Latic acid, the main metabolic endproduct of *Lactobacillus* would improve the bioavailability of trace minerals (Famularo et al., 2005). Nath et al. (2018) reported that *Butyricimonas* is correlated with lipid metabolism and positively associated with the production of high-density lipoprotein for an individual’s health. Meanwhile, Enterococcus is known to produce enterocins to improve energy and protein utilization associating with higher growth rates, which is in accordance with this studies performance results (Fisher and Phillips, 2009). The predominant microbiota results in this study suggested that dietary supplemented with coated trace minerals could improve the enrichment of beneficial bacterium in duck cecum to improve and maintain gut health.

The contamination of metals in the environment is an important cause of stress and detrimental health effects. Recent studies showed that microorganisms inhabiting the gastrointestinal tract could provide essential services to reduce environmental contamination (Brila et al., 2021). To explore the relationships between the extraction of trace minerals and gut microbiota, RDA analysis was conducted in the present study. Intestinal *Fusobacterialse*, *Clostridiales*, *Turicibacterales*, and *Bifidobacteriales* had negative relationships with fecal trace mineral contents. Cheng et al. (2020) found that cecal *Bifidobacteriales* and *Clostridiales* were altered by copper, which is similar to the findings in the present study. Trace minerals, such as Cu and Zn could be used by *Bifidobacteriales* to produce probiotic substrates (Ionescu et al., 2009), thus it has a negative relationship with excreted trace minerals. On the other hand, *Bifidobacteriales* has a crucial role in body health and was seen as a potential beneficial body service provider (Zhang et al., 2016). Spearman’s correlation analysis also showed that fecal ion metals had a significant negative relationship with cecal *Coprobacillus*, especially Se. *Coprobacillus* was a Gram-positive bacteria, closely associated with *Clostridum ramosum* (Kageyama and Benno, 2000), and its colonization could be improved by *Enterococcus* (Stein et al., 2013). Thus, as coated trace minerals may improve *Enterococcus* colonization in duck cecum, this might have the ability to improve the growth of *Coprobacillus* to reduce excretion of trace minerals.

The predicated KEGG analysis results showed that coated trace mineral groups significantly upregulated pathways related to carbohydrate metabolism and stimulated glycerolipid metabolism and membrane transport. The upregulation in carbohydrate metabolism could well support the better growth performance. Yin et al. (2021) reported that coated trace mineral could improve lipid metabolism, which might be associated with the present result. Meanwhile, ion metals, such as Zn and Cu, were related with microbiota function (van Kuijk et al., 2021). Inorganic trace metals would result in lower bioavailability and, thus, greater competition between intestinal microbiota and host (Reed et al., 2015). While coated trace minerals might improve the stability and absorption of minerals *via* the intestinal brush border, they may also be more effectively associated with the upregulation of microbiota transporter systems. Consequently, dietary supplementation with coated trace minerals could stimulate microbial barrier function.

## Conclusion

In conclusion, the present study suggested that coated trace minerals enhanced the growth of meat ducks. Coated trace minerals used in duck diets could reduce the risk of environmental contamination from fecal minerals without affecting performance. Additionally, 500 mg/kg of coated trace mineral had a similar effect on duck serum trace minerals and tissue ion metal deposition relative to 1,000 mg/kg of inorganic trace minerals. Coated trace minerals had a potential to improve bioavailability of ion metals and the colonization of probiotic microbiota, such as *Lactobacillus*, *Butyricimonas*, and *Enterococcus*, in the cecum to protect the intestine microbial barrier and maintain gut health.

## Data Availability Statement

The datasets presented in this study can be found in online repositories. The names of the repository/repositories and accession number(s) can be found at: https://www.ncbi.nlm.nih.gov/, PRJNA787257.

## Ethics Statement

The animal study was reviewed and approved by Experimental Animal Committee of Shenyang Agricultural University (permit number 202106037).

## Author Contributions

DY: data analysis and writing original draft. FZ: data curation and investigation. AM: review and edit draft. YK: investigation. WL, FL, YuZ, and RZ: data curation. YoZ and SZ: supervision. All authors contributed to the article and approved the submitted version.

## Funding

This work was supported by the China Postdoctoral Science Foundation (no. 2021M702299).

## Conflict of Interest

FZ was employed by the company Yichun Tequ Feed Company and WL and YK were employed by the company Fujian Syno Biotech Co., Ltd.

The remaining authors declare that the research was conducted in the absence of any commercial or financial relationships that could be construed as a potential conflict of interest.

## Publisher’s Note

All claims expressed in this article are solely those of the authors and do not necessarily represent those of their affiliated organizations, or those of the publisher, the editors and the reviewers. Any product that may be evaluated in this article, or claim that may be made by its manufacturer, is not guaranteed or endorsed by the publisher.
