# Supplementary material for: Comparison of Coated and Uncoated Trace Minerals on Growth Performance, Tissue Mineral Deposition, and Intestinal Microbiota in Ducks
Source: Front Microbiol. 2022 Apr 12;13:831945. doi: 10.3389/fmicb.2022.831945 (PMC9039745; doi:10.3389/fmicb.2022.831945)
Supplement: Supplementary file 1 [file Table_1.DOCX]

| Table S1. The contents of trace minerals (coated and uncoated) at each trace mineral supplement level, mg/kg. | | | | | | |
| --- | --- | --- | --- | --- | --- | --- |
| Level | Se | Cu | Zn | Fe | Mn | I |
| 300mg/kg | 0.24 | 3.9 | 36 | 42 | 36 | 0.3 |
| 500mg/kg | 0.40 | 6.5 | 60 | 70 | 60 | 0.5 |
| 1000mg/kg | 0.80 | 13.0 | 120 | 140 | 120 | 1.0 |
