# Supplementary material for: Comparison of Coated and Uncoated Trace Minerals on Growth Performance, Tissue Mineral Deposition, and Intestinal Microbiota in Ducks
Source: Front Microbiol. 2022 Apr 12;13:831945. doi: 10.3389/fmicb.2022.831945 (PMC9039745; doi:10.3389/fmicb.2022.831945)
Supplement: Supplementary file 2 [file Table_2.DOCX]

Fig S1. Differentially top 20 microbiota at family levle between diets supplemented with different level of coated and uncoated trace minerals. CoL, 300mg/kg coated trace mineral treatment; CoM, 500mg/kg coated trace mineral treatment; CoH, 1000mg/kg coated trace mineral treatment; UCoL, 300mg/kg uncoated trace minerals treatment; UCoM, 500mg/kg uncoated trace minerals treatment; UCoH, 1000mg/kg uncoated trace minerals treatment.
